# Deletions within *COL11A1* in Type 2 stickler syndrome detected by multiplex ligation-dependent probe amplification (MLPA)

**DOI:** 10.1186/1471-2350-14-48

**Published:** 2013-04-26

**Authors:** Raymon Vijzelaar, Sarah Waller, Abdellatif Errami, Alan Donaldson, Teresa Lourenco, Marcia Rodrigues, Vivienne McConnell, Gregory Fincham, Martin Snead, Allan Richards

**Affiliations:** 1 MRC-Holland, Amsterdam, The Netherlands; 2 East Anglian Regional Genetics Laboratory, Addenbrooke’s Hospital, Cambridge University Hospitals NHS Trust, Cambridge, UK; 3 Genetic Medicine, St Mary’s Hospital, Manchester, UK; 4 Clinical Genetics, University Hospitals Bristol, NHS Trust, Bristol, UK; 5 Department of Medical Genetics, Hospital de Dona Estefânia, Lisbon, Portugal; 6 Northern Ireland Regional Genetics Service, Belfast City Hospital, Belfast, UK; 7 Vitreoretinal Service, Department of Ophthalmology, Addenbrooke’s Hospital, Cambridge University Hospitals NHS Trust, Cambridge, UK; 8 Department of Pathology, University of Cambridge, Cambridge, UK

**Keywords:** *COL11A1*, MLPA, Molecular analysis, Stickler syndrome

## Abstract

**Background:**

*COL11A1* is a large complex gene around 250 kb in length and consisting of 68 exons. Pathogenic mutations in the gene can result in Stickler syndrome, Marshall syndrome or Fibrochondrogenesis. Many of the mutations resulting in either Stickler or Marshall syndrome alter splice sites and result in exon skipping, which because of the exon structure of collagen genes usually leaves the message in-frame. The mutant protein then exerts a dominant negative effect as it co-assembles with other collagen gene products. To date only one large deletion of 40 kb in the *COL11A1*, which was detected by RT-PCR, has been characterized. However, commonly used screening protocols, utilizing genomic amplification and exon sequencing, are unlikely to detect such large deletions. Consequently the frequency of this type of mutation is unknown.

**Case presentations:**

We have used Multiplex Ligation-Dependent Probe Amplification (MLPA) in conjunction with exon amplification and sequencing, to analyze patients with clinical features of Stickler syndrome, and have detected six novel deletions that were not found by exon sequencing alone.

**Conclusion:**

Exon deletions appear to represent a significant proportion of type 2 Stickler syndrome. This observation was previously unknown and so diagnostic screening of *COL11A1* should include assays capable of detecting both large and small deletions, in addition to exon sequencing.

## Background

Stickler syndrome (OMIM # 108300, 604841, 184840), first described in 1965 [1] most commonly occurs as an autosomal dominantly inherited connective tissue disorder. It is caused by mutations in collagen genes that encode the heterotypic collagen fibrils present in cartilage and vitreous, namely *COL2A1*, *COL11A1* and *COL11A2*[2-4]. These genes encode different collagen α chains that co-assemble into either a type II collagen homotrimer (all three chains being products of the *COL2A1* gene), or a type XI collagen heterotrimer that can contain products from all three genes [5]. We have recently demonstrated the use of MLPA to detect large deletions encompassing the whole *COL2A1* gene [6] but no equivalent system was available for *COL11A1*. The *COL11A1* gene is approximately 250 kb in length, located on chromosome 1p21 and comprises in total 68 exons. To date, the majority of mutations in *COL11A1* affect the consensus splice sites of exons encoding the collagen helical domain, and those that have been analyzed by RT-PCR have all indicated exon skipping. Because skipping of these exons leaves the message in-frame and the Gly-Xaa-Yaa repeating collagen motif in register, this results in mutant collagen α chains capable of co-assembly with α chains synthesized from the normal genes, resulting in a dominant negative effect [6,7]. Only one large deletion (~40 kb) encompassing exons 32–43 has previously been described [8]. However, this was identified by analyzing mRNA via RT-PCR, and so the commonly used strategy of amplifying relatively small genomic regions and exon sequencing means that similar mutations could easily remain undetected. Currently the frequency of such large deletions is unknown [9]. The aim of this study was to detect *COL11A1* copy number alterations using a multiplex ligation-dependent probe amplification (MLPA) assay [10]. Here we present six examples of Stickler syndrome which display exon deletions of *COL11A1* found by this technique, that were not detected by exon sequencing alone.

## Methods

### MLPA

During a 2 year period between mid 2010 and mid 2012, patient samples, submitted to the Cambridge Regional, Molecular Genetics Diagnostic Service UK, were tested for genomic rearrangements in the *COL11A1* gene using MLPA, as previously described [10]. Due to the large number of exons in *COL11A1* and the limitation of the number of probes which can be included in an MLPA assay, two different assays (P381 and P382) were developed.

**Figure 1:**
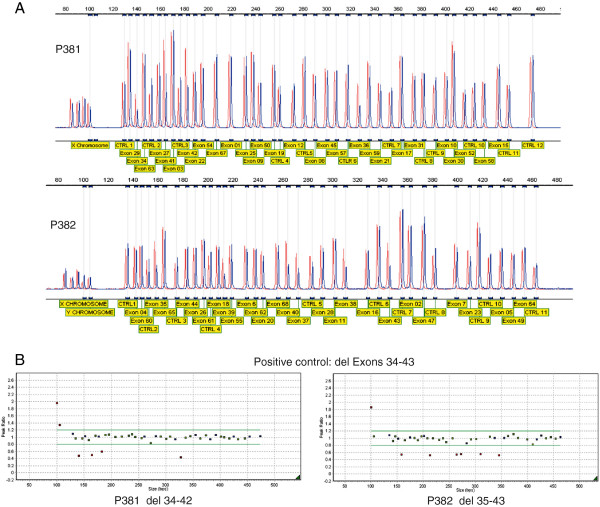
**Validation of the MLPA probe sets.** In addition to 50 normal DNAs the probe sets were used to analyze a positive control with a deletion of exons 32–43. The top two panels (**A**) show the difference in peak height between a normal control (red) and the positive control (blue) for both MLPA kits. The size and target of each product is also indicated. This data is also represented in the bottom two panels (**B**), where blue squares represent control amplicons, green squares are probes for *COL11A1* that fall within peak ratios of 1.2 -0.8 of a normalised control. Red squares fall above or below peak ratios of 0.8, indicating a duplicated or deleted region. The probes for the X and Y chromosomes (far left) may differ depending upon the gender of the DNA control. Probes for *COL11A1* exons 32 and 33, which are known to be within the deleted region, are not included in either kit.

The *COL11A1* gene is alternatively spliced and no single transcript contains all 68 exons, these have also been numbered 1–67 [7] with the alternatively spliced exons 6 and 7 being labeled 6A and 6B. The most commonly used cDNA reference sequence used for reporting mutations is NM_001854.3, which contains all of the 68 exons except exon 7 (also referred to as exon 6B). An alternative transcript NM_080629.2 includes exon 7 but excludes exon 6 (alternatively referred to as exon 6A). Here we use the exon numbering of 1–68, and the cDNA reference sequence NM_001854.3 to describe the effect on the cDNA and protein, where c.1 refers to the A of the initiating ATG codon (unless otherwise stated). The MLPA assays were designed by MRC-Holland (Amsterdam, the Netherlands) and comprise in total fifty seven *COL11A1* exon specific probes. Twenty two probes were located at other chromosomal locations and served as reference probes. The exon specific probes in each kit are detailed in Figure 1A and also on the manufacturer’s web site (http://www.mrc-holland.com/WebForms/WebFormMain.aspx). No probes are present for exons 13, 14, 24, 32, 33, 46, 48, 51, 53, 56 and 66 of the *COL11A1* gene. Note that the MLPA kits and the cDNA reference sequence NM_001854.3 names the exons 1–68. Both MLPA assays contain 9 quality control probes to assess DNA denaturation and DNA quantity, and also for the X and Y chromosome. MLPA was performed according to manufacturer’s protocol [http://www.mrc-holland.com/WebForms/WebFormMain.aspx]. Fragment analysis was conducted using GeneMarker software [http://www.softgenetics.com; Soft Genetics, State College, PA, USA]. Validation of the two sets of MLPA probes for *COL11A1* was established by analyzing DNA from 50 normal controls as well as two control DNAs from patients with known deletions. The positive controls were a deletion of exons 32–43 [8] that was detected by both sets of probes and a 27 bp deletion in exon 44 [6] that was detected by only one of the probe sets. These normal and positive controls showed that the MLPA assays were performing as expected, data for the large deletion is shown in Figure 1. The two MLPA probes sets were then used in addition to routine exon sequencing when analyzing patients with Stickler syndrome.

Long range PCR was used to determine if certain exons not covered by the MLPA probe set (due to the limited number of probes in each assay) were included or absent from the deleted regions. Patient DNA was amplified using either Sequalprep [Invitrogen, Life Technologies Ltd, Paisley PA4 9RF, UK] or a long PCR enzyme mix [Thermo Fisher scientific; Hemel Hempstead, HP2 7GE, UK] along with primers known to be outside of the deleted area. Typical cycling conditions were 94°C 3 min followed by 10 cycles consisting of 94°C 30 sec, 55°C 30 sec, 68°C 8 min, followed by 25 cycles consisting of 94°C 30 sec, 55°C 30 sec, 68°C 8 min + 20 sec/cycle. The elongation time was extended to 14 min when trying to amplify particularly large regions of DNA.

## Case presentations

**Figure 2:**
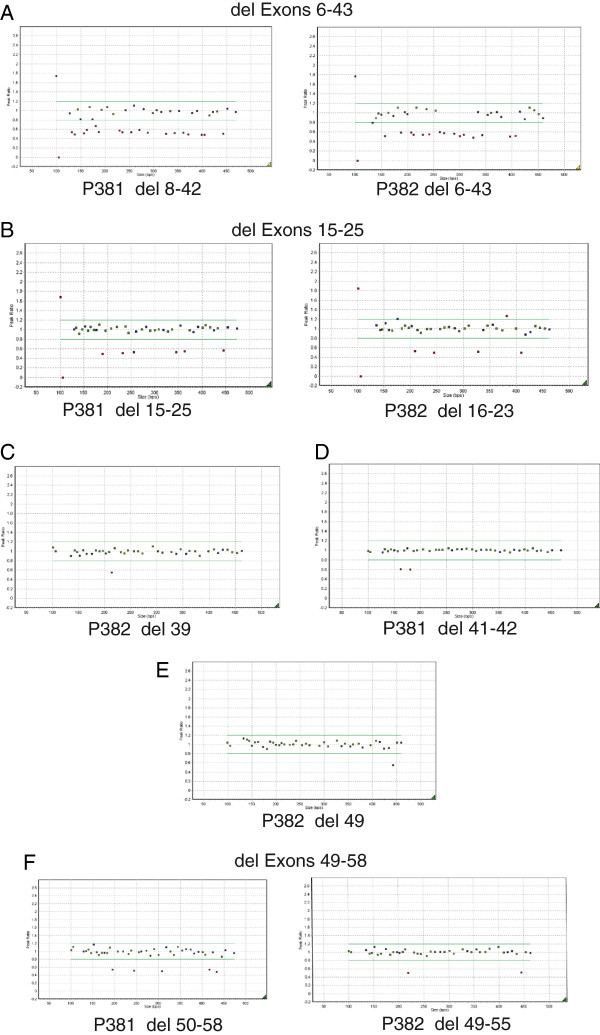
**MLPA Detects Multi Exon Deletions.** DNA from the six cases (**A**-**F**) were analyzed by MLPA. Blue squares represent control amplicons. Green squares are probes for *COL11A1* that fall within peak ratios of 1.2 -0.8 of a normalised control. Red squares fall above or below peak ratios of 0.8, indicating a duplicated or deleted region. The probes for the X and Y chromosomes (far left) may differ depending upon the gender of the DNA control.

Deletions were detected in six patients in whom no mutation was found by exon sequencing and are illustrated in Figure 2.

### Patient 1

The proband (male) was examined at age 7 months due to Pierre Robin sequence. The patient was not myopic and there was no sensorineural hearing loss. However there was a family history of myopia and retinal detachment. Examination of the mother showed her to be a low myope, with a beaded vitreous architecture. One probe set (P382) detected a deletion of exons 6–43, and the other probe set (P381) a deletion of exons 8–42, (Figure 2A) resulting in a minimal deleted region of exons 6–43 [c.781-3276, p.Tyr261-Pro1092] As exons 5 and 44 are included within both probe sets no further analysis was performed. In the alternative transcript NM_080629.2 this deletion would encompass c.781-3312, p.Lys261-Pro1104.

### Patient 2

**Figure 3:**
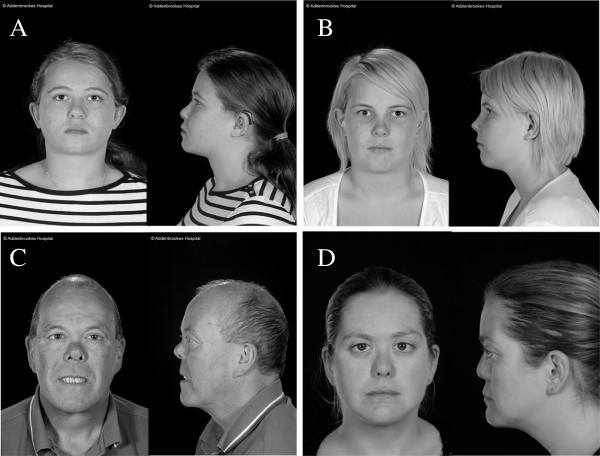
**Facial Photographs of patients with deletions in the *COL11A1 *gene.** Two sisters (**A** and **B**) with a deletion of *COL11A1* exons 14–25. A father and daughter (**C** and **D**) with a deletion of *COL11A1* exon 49.

The proband (female) was seen at age 14 years and was found to have abnormal vitreous gel architecture, with a beaded phenotype. She had a flat midface with nasal bridge hypoplasia, anteverted nares and micrognathia (Figure 3A). There was both sensorineural and conductive hearing loss and she used hearing aids with good effect. Radiographs showed irregularity of end plates of several of the cervical, thoracic and lumbar vertebrae. There was also some anterior wedging of multiple vertebral bodies. One MLPA probe set (P381) detected a deletion of exons 15–25, and the other probe set a deletion of exons 16–23 (P382, Figure 2B). Because the two sets of probes did not cover exons 13 and 14, DNA from this patient was amplified with primers upstream of exon 12 and downstream of exon 26. The amplified product was sequenced which demonstrated that exon 14 was also contained within the deleted region [c.1486-2142, p.Pro496-Lys714] but that exon 13 was not deleted. The mutation was also present in her sister (Figure 3B), her father and grandfather are also thought to be clinically affected but their DNA has not been analyzed.

### Patient 3

The female proband (Figure 3D) was examined at age 29. She had previously had a left retinal detachment. The right eye had an abnormal vitreous architecture with a beaded phenotype and a quadrantic lamellar cataract. There was a family history of retinal detachment on her paternal side. MLPA analysis (Figure 2E) indicated a deletion of exon 49 [c.3655-3708, p.Gly1219-Asp1236]. As exon 48 is not covered by the two probe sets, long range PCR and sequencing using primers encompassing exons 48–50 was performed, which confirmed deletion of exon 49 and that exon 48 was not included in the deleted region. The mutation was also confirmed in her father (Figure 3C).

### Patient 4

A two and a half year old male proband was found to be highly myopic with a facial phenotype (maxillary hypoplasia and a flattened nasal bridge) suggestive of Stickler syndrome. No sensorineural hearing loss was demonstrated, but there was a history of recent glue ear. Examination revealed a beaded vitreous phenotype and a high-arched palate, but no midline clefting. Deletions of exons 41 and 42 [c.3025-3168, p.Gly1009-Val1056] were detected by MLPA (Figure 2D), as probes for exons 40 and 43 are included in the two probe sets no further analysis was required.

Two additional mutations were detected in DNA from other patients with Stickler syndrome (Figure 2C and F). These were a deletion of a single exon, 39 [c.2863-2916, p.Gly955-Gln972] and a deletion of exons 49–58 [c.3655-4302, p.Gly1219-Met1434]. In this latter case it could not be confirmed whether or not exon 48 was included in the deleted region, probably due to the large size of the region needed to be amplified by long range PCR.

## Conclusion

Here we have described the design and utilization of MLPA probes sets that cover 57 of the 68 exons of the *COL11A1* gene, and have detected six novel deletions in patients with Stickler syndrome that would have been overlooked by exon sequencing alone. All six leave the message in frame and the repeating Gly-Xaa-Yaa amino acid sequence of the collagen triple helical region in register and the C-propeptide region, through which type XI collagen chains assemble into heterotrimers, intact. So these mutant molecules are capable of co-assembling with normal procollagen and consequently disrupt their normal function. The six deletions described here were not detected by exon sequencing. During the same period that these mutations were detected, twenty two other *COL11A1*mutations were identified by exon sequencing in the Cambridge Regional Molecular Genetics Diagnostic Laboratory, UK. [http://www.cuh.org.uk/addenbrookes/services/clinical/genetics/genetics_labs/contacts.html]. So, although the exact frequency of large deletions in *COL11A1* remains uncertain, they would appear to represent a significant proportion of type 2 Stickler syndrome cases, and laboratories offering diagnostic sequencing of *COL11A1* should include a protocol capable of detecting deletions of various sizes.

As next generation sequencing becomes a standard method of mutation detection in diagnostic service laboratories, it may be possible to recognize gene deletions by identifying regions where there is homozygosity and a reduction in read depth. This may be more achievable using capture methods rather than sequencing PCR products, where identification of large deletions may be dependant upon primer design. In the short term deletions identified by next generation sequencing may need to be verified by another method to confirm the mutation.

Five of the six deletions were confined to the collagen triple helical region of the molecule. One other also included the alternatively spliced exons 6, 7 and 9 which form part of the variable N-propeptide. However, the individuals with this region deleted did not appear clinically different from other patients with type 2 Stickler syndrome. This probably reflects that, as with the other multi-exon and single-exon deletions, the mutant α chains co-assemble with normal α chains synthesized from the *COL2A1*, *COL11A1* and *COL11A2* genes, forming an abnormally folded molecule that undergoes intracellular retention and degradation [11].

## Consent

Written informed consent was obtained from the patients for publication of these case reports and any accompanying images. A copy of the written consent is available for review by the Editor of this journal.

## Competing interests

Raymon Vijzelaar and Abdellatif Errami are employees of MRC-Holland, manufacturer of commercially available MLPA probemixes. Other authors: No conflict of interests.

## Authors’ contributions

The MLPA probes were designed by RV and AE, the assay was validated and implemented by SW and AR. The patients were clinically assessed by AD, TL, MR, VM, GF and MS. The manuscript was written by RV and AR, read and edited by the other authors. All authors have read and approved the final manuscript.

## Pre-publication history

The pre-publication history for this paper can be accessed here:

http://www.biomedcentral.com/1471-2350/14/48/prepub
